# 

**Published:** 1905-11-11

**Authors:** 


NOTICE TO CORRESPONDENTS.

All MSS., Letters, Books for Review, and other matters intended for the Editor should be addressed THE EDITOR, "THE HOSPITAL," THE HOSPITAL BUILDINGS, 28 & 29 SOUTHAMPTON STREET, STRAND, W.C.

The Editor cannot undertake to return rejected MS., even when accompanied by stamped directed envelope.

**Notice to Advertisers and Subscribers.**

All Advertisements, Orders for Copies of THE HOSPITAL, and Business Communications should be addressed to **THE MANAGER (Not to the Editor),** THE HOSPITAL, 28 & 29 Southampton Street, Strand, London, W.C.

The Cost of Subscription, post free, is at follows:—Per week, 3½d.; per quarter, 3s. 3d.; per half-year, 6s. 6d.; per annum, 13s. Foreign and Colonial:—Per annum, 19s. 6d., payable, in all cases, in advance.

**NOTICE.—Vol. XXXVII. of "The Hospital" October 1904 to March 1905), in handsome cloth binding, will shortly be on sale at the office of this Journal, price 10s. 6d. Cases for binding the Numbers contained in this Volume 2s. Index to Vol. XXXVII., 3½d., post free.**

**The Monthly Part for November is now ready. Price 1s., by post, 1s. 3d.**

### BOOKS RECEIVED.

Baillière, Tindall, and Cox.

"Nodal Fever." By A. A. Lendon, M.D.

H. Kimpton.

"Essentials of Medical Electricity." By R. Morton, M.D.

The Scientific Press, Limited.

"Medical Electricity and Light Treatment." By Kate Neale.

"Nursing Hints to Probationers." By M. H. Annesley Voysey.

"Nursing of Sick Children." By J. Burnet, M.D.

Smith, Elder, and Co.

"Ellis's Demonstrations of Anatomy." Edited by C. Addison, M.D.

J. Wright and Co., Bristol.

"Golden Rules of Sick Nursing."

### Medical Appointments.

**Walter Herbert Barker,** M.R.C.S.Eng., L.R.C.P.Lond., Medical Superintendent at the Hospital for the Insane, Kew, Victoria, Australia. **Charles Edward Barnard,** M.D.Aberd., Acting Officer of Health of the Shire of Creswick, Victoria, Australia. **George Biggs,** M.B., B.S., L.S.A., Clinical Assistant to the Chelsea Hospital for Women. **G. Black,** M.B., B.S.Aberd., Certifying Surgeon under the Factory and Workshop Act for the Hailsham District of the County of Sussex. **R. J. Blackham,** L.R.C.P.&S.Edin., L.F.P.S.Glasg., D.P.H. Lond., an examiner to the St. John Ambulance Association. **Arthur H. Burgess,** F.R.C.S.Eng., M.B., M.Sc.Vict., Surgeon to the Manchester Children's Hospital, Pendlebury. **E. Temple Curran,** Medical Officer for the Second District, by the St. Columb (Cornwall) Board of Guardians. **W. Dalsh,** M.D.Melb., Honorary Medical Officer of the Dubbo Hospital, New South Wales. **D. Fletcher,** L.R.C.P. & S.Edin., L.F.P.S.Glas., Certifying Surgeon under the Factory and Workshop Act for the Harris District of the County of Inverness. **J. C. Gardner,** M.B., B.S.Cantab., Certifying Surgeon under the Factory and Workshop Act for the Amersham District of the County of Buckingham. **Clarence George Godfrey,** M.R.C.S.Eng., L.R.C.P. & S.Edin., Medical Superintendent at the Hospital for the Insane, Ararat, Victoria, Australia. **Cooper R. Higham,** L S.A., Assistant Medical Officer in the Electro-Therapeutic Department, University College Hospital. **Forbes Kinnear,** M.B., Ch.B.Aberd., Certifying Surgeon under the Factory and Workshop Act for the Royton District of the County of Lancashire, and Surgeon to the Police for Royton and Shaw, near Oldham. **Cecil J. R. MacFadden,** M.D. Edin., Honorary Assistant Medical Officer to the Hampstead General Hospital. **Anwyl Charles Matchett,** M.B., C.M. Edin., District Medical Officer, by the Bridgwater (Somerset) Board of Guardians. **J. Morris,** M.R.C.S., L.R.C.P.Lond., Resident House Surgeon to the British Hospital, Buenos Ayres. **William Lowell Mullen,** M.D.Melb., Medical Superintendent at the Hospital for the Insane, Yarra Bend, Victoria, Australia. **George Lathrop Murray,** M.B., M.Ch.Syd., Government Medical Officer and Vaccinator at Portland, New South Wales. **John Aloysius O'Brien,** M.B., Ch.M.Glasg., Inspector of Institutions under the Inebriates Act, 1904, for Victoria, Australia. **Arthur Geoffrey Owen,** M.B.Melb., Officer of Health of the Shire of Hampden, West Riding, Victoria, Australia. **Frank Robinson,** M.D.Vict., D.P.H., Assistant to the County Medical Officer, West Riding of Yorks. **G. W. Ross,** M.R.C.S., L.R.C.P.Lond., Pathologist to the City of London Hospital for Diseases of the Chest, Victoria Park, E. **Frank E. Scrase,** F.R.C.S.Eng., D.P.H., Honorary Assistant Medical Officer to the Hampstead General Hospital. **J. W. Somerville,** M.D.Edin., Certifying Surgeon under the Factory and Workshop Act for the Galashiels District of the County of Selkirk. **W. H. Maxwell Telling,** M.D.,B.S.Lond., M.R.C.P.Lond., Honorary Assistant Physician to the General Infirmary, Leeds. **R. J. Waugh,** M.R.C.S.Eng., L.R.C.P. Lond , Assistant Medical Superintendent at the Fulham Infirmary, Hammersmith, W. **Robert Neville Woodside,** M.B., Ch.B.Melb., Officer of Health of the Shire of Gordon (eastern portion), Victoria, Australia. **Thomas Nisbett Wright,** L.R.C.S.& P.Irel., L.M., Government Medical Officer and Vaccinator at Gloucester, New South Wales.

### Handbooks for Midwives and District Nurses

***Complete catalogue sent post free on application***

The Book for the C.M.B. Exam.

**A COMPLETE HANDBOOK OF MIDWIFERY.**

By J. K. WATSON, M.D. **6/-** net, **6/4** post free.

Profusely Illustrated with 143 Diagrams.

This book has been **compiled in accordance with the requirements of the Central Midwives Board,** and will prove not only the best text-book for those entering for the next exam., but is so complete as to be of greatest value to the Qualified Midwife for immediate reference in cases of emergency.

The Midwives Act Explained.

**HOW TO BECOME A CERTIFIED MIDWIFE.**

By Dr. APPEL. **1/-** net; **1/1** post free.

This book gives a very clear and complete explanation of the Midwives Act, and a copy should be in the possession of all Midwives and those nurses desirous of taking up the special branch.

**INSTRUCTIONS TO MIDWIVES.**

Compiled by the Manchester Midwives' Committee.

Crown 8vo. cloth, **6d.** net, **7d.** post free.

Midwives will find this little work exceedingly useful as it explains simply and concisely what should be done in accordance with the rules of the Midwives Act.

A Valuable Reference Book for Practising Midwives.

**A PRACTICAL HANDBOOK OF MIDWIFERY.**

By FRANCIS W. HAULTAIN, M.D.

Illustrated, **6/-** net; **6/3** post free.

The information contained in this work is arranged in such a way as to render it of greatest practical value as a work of reference.

An Important New Work.

**SIMPLE SANITATION:**

**The Practical Application of the Laws of Health to Small Dwellings.**

By M. LOANE,

Superintendent of District Nurses, Portsmouth.

Limp cloth, **1/-** net, **1/2** post free.

With an Introductory Chapter on Sanitary Legislation by Dr. A. MEARNS FRASER, Medical Officer of Health, Portsmouth.

This work should prove of great practical assistance to Midwives and District Nurses, as it contains Chapters on Sanitary Legislation, Water, Air and Ventilation, Drainage and Disposal of Refuse, Household Management, Personal Hygiene, Disinfectants, &c.

Dictionary of Terms used in Medicine, Midwifery and Nursing.

**THE NURSES' PRONOUNCING DICTIONARY.**

By HONNOR MORTEN. **2/-** post free.

New Edition, Revised by MARY I. BURDETT.

This Dictionary has been used by Nurses for many years, and the fact that it has been recently revised and the phonetic pronunciations added by Miss M. I. Burdett should greatly enhance its value as a work of reference. Its size enables it to be carried comfortably in the apron pocket, which **is a** strong point in its favour.

A Useful and Handy Guide for the Apron Pocket.

**THE MIDWIVES' POCKET BOOK.**

By HONNOR MORTEN. **1/-** post free.

The many thousands of copies that have been sold of this handbook proves the great usefulness of this very helpful little manual.

How to Bandage.

**PRACTICAL GUIDE TO SURGICAL BANDAGING AND DRESSINGS.**

By WM. JOHNSON SMITH, F.R.C.S. **2/-** post free.

A useful guide to Bandaging and Dressing, written especially for Nurses by a surgeon of great experience. The work is up to date and the information thoroughly reliable.

A Practical Book on District Nursing.

**PRACTICAL HINTS ON DISTRICT NURSING.**

By AMY HUGHES,

General Superintendent-Elect Queen Victoria's Jubilee Nurses.

**1/-** post free.

A work on District Nursing by the foremost District Nurse in the United Kingdom.

Children.

**NURSING OF SICK CHILDREN.**

By Dr. BURNET. **1/-** net, **1/1** post free.

District Nurses and Midwives will find this a most useful handbook.

Case-Books.

**THE "SIMPLEX" DISTRICT NURSES' CASE-BOOK.** **6d.** net; **7d.** post free.

Contains space for fifty cases, ruled and printed, bound in cloth boards.

**THE POCKET CASE-BOOK FOR DISTRICT AND PRIVATE NURSES.**

By a PHYSICIAN.

Demy 16mo. cloth boards, **1/-** post free; in handsome leather, gilt, **1/6** net, **1/7** post free.

**The Scientific Press, Ltd.**

**Publishers of Nursing Text-Books, Charts, and Case-Books of all Descriptions.**

**28 & 29 SOUTHAMPTON STREET, STRAND, LONDON, W.C.**

